# Association between body roundness index and suicidal ideation in U.S. adults: A cross-sectional study

**DOI:** 10.1016/j.pmedr.2025.103018

**Published:** 2025-02-21

**Authors:** Pincheng Luo, Omar M. Alruwaili, Huanlin Zhou, Yanxue Lian

**Affiliations:** a School of Medicine, University of Galway, Galway, Ireland; b School of Medicine, University College Dublin, Ireland; c School of Kinesiology, University of Michigan, Ann Arbor, MI, USA

**Keywords:** Body roundness index, Body shape, Suicidal ideation, Metal health, Public health

## Abstract

**Purpose:**

The relationship between the body roundness index (BRI) and suicidal ideation has not been previously examined. This study aimed to investigate this association through a cross-sectional analysis.

**Method:**

The data used in this study was extracted from the National Health and Nutrition Examination Survey ranging from 2011 to 2012 to 2017–2018. The BRI was calculated from body measurement data, while a questionnaire was used to assess suicidal ideation. Weighted multivariable logistic regression analysis, weighted stratified analysis, and smooth curve fitting were conducted to assess the relationship between the BRI and suicidal ideation. **Results:** A total of 12,878 participants were included in the study with 441 of them reported experiencing suicidal thoughts. After adjusted for all covariates, a one-unit increase in BRI was associated with a 5 % greater likelihood of having suicidal thoughts [1.05 (1.00, 1.10)]. Additionally, BRI was categorized into quartiles, and individuals in the highest quartile were 1.52 times as likely to experience suicidal thoughts compared to those in the lowest quartile [1.52 (1.03, 2.25)]. There were no interaction effects between BRI and suicidal ideation for any subgroups stratified by demographics.

**Conclusion:**

The present study indicated that a greater BRI was significantly associated with higher levels of suicidal ideation. The findings underscore the importance of considering BRI as a potential indicator for identifying individuals at elevated risk of suicidal ideation. The study also emphasizes the need for a shift towards a weight-inclusive approach in public health, which can help reduce societal pressures and mitigate the psychological impact of weight stigma.

## Highlights

• Each unit rise in body roundness linked to 5 % higher odds of suicidal thoughts.
• Body roundness index may help identify those at higher risk of suicidal thoughts.
• Weight-inclusive approaches promote mental health by reducing societal pressures.
• Addressing weight stigma can mitigate psychological distress linked to suicide risk.

## 1 Introduction

Suicidal ideation encompasses thoughts, ideas, or considerations about ending one's own life, ranging from the belief that one would be better off dead to detailed planning of suicide (World Health Organization, 2024). Suicide is a major public health concern that greatly increases the global burden of disease and mortality (World Health Organization, 2024). According to the World Health Organization, more than 700,000 people take their own lives annually, ranking suicide among the top causes of death globally (World Health Organization, 2024). In America, suicide is also a significant public health issue. The Centers for Disease Control and Prevention (CDC) reported that approximately 49,449 Americans died by suicide in the year 2022, representing approximately 7.1 % of the global suicide population (Centers for Disease Control and Prevention, 2024). Research indicates that suicidal ideation is more prevalent than actual suicide attempts, making it a crucial target for prevention efforts (Klonsky et al., 2021). Moreover, suicidal thoughts often precede suicide attempts, underscoring their significance as a critical warning sign. Despite extensive efforts in the political, social, and medical spheres to promote well-being and prevent suicide, rates have been steadily increasing over the past two decades in America, from 10.4 per 100,000 in 2000 to 14.1 per 100,000 in 2021 (Centers for Disease Control and Prevention, 2024). This trend emphasizes the urgent need for a thorough investigation of the suicidal ideation.

Recent research has highlighted the complex nature of suicidal ideation, which arises from a combination of psychological, biological, and environmental factors (Klonsky et al., 2016). Mental health disorders, such as post-traumatic stress disorder, depression, and bipolar disorder, are strongly linked to suicidal ideation (Klonsky et al., 2016). Additionally, biological factors, such as imbalances in brain chemicals, particularly reduced serotonin levels, are associated with increased impulsivity and aggression, heightening the risk of suicidal thoughts (Pandey, 2013). Environmental stressors, including financial difficulties, unemployment, and a lack of social support, can contribute to suicidal ideation (Safaei Lari and Emamgholipour, 2023). Physical health conditions, such as chronic pain and terminal illnesses, are also significant factors (Racine, 2018). Experiencing obesity, often underestimated, significantly impacts mental health. Experiences of weight-related discrimination, stigma, and internalized biases can lead to heightened stress, adverse behavioral shifts, reduced healthcare engagement, and feelings of social isolation, all of which may contribute to an increased risk of suicidal ideation (Brochu et al., 2018; Hunger et al., 2020; Pearl and Puhl, 2018).

Previous research has utilized various obesity metrics to investigate the relationship between obesity and suicidal ideation. A cross-sectional study identified a positive association between an elevated weight-adjusted waist index (WWI) and increased suicidal thoughts (Guo et al., 2024a). This finding aligns with another study using body mass index (BMI) as the metric, which reported significant associations between both overweight [1.10 (1.01, 1.20)] and obesity [1.17 (1.01, 1.35)] with heightened suicidal ideation in adolescents (Zhang et al., 2022). However, a similar study by Graham and Frisco (2022) involving young American adults found no significant relationship between BMI and suicidal ideation (Graham and Frisco, 2022). Although a review of the literature suggests that more studies report a positive association between obesity and suicidal ideation than those reporting negative or null findings, the connection remains inconclusive and warrants further investigation.

While BMI is commonly used to assess individuals with obesity and is calculated as weight in kilograms divided by height in meters squared (Nevill et al., 2006), it has notable limitations. These include its inability to distinguish between fat and muscle, disregard for fat distribution, and lack of accuracy across different demographic groups (Nevill et al., 2006). In response to these limitations, WWI has emerged as a new metric and has been linked to increased suicidal ideation (Guo et al., 2024b). WWI provides greater accuracy in assessing both lean muscle and fat mass, as it has been shown to correlate positively with fat mass and negatively with muscle mass (Kim et al., 2023). Nonetheless, as Wang et al. highlighted, the reliability of obesity indicators may be compromised when height is not adequately considered, particularly for individuals who are exceptionally short or tall (Wang et al., 2022).

The body roundness index (BRI), introduced by Thomas et al. in 2013, is an innovative geometric model that combines waist circumference and height to assess body shape and central adiposity in individuals (Thomas et al., 2013). Studies across various populations and ethnic groups have validated the BRI as a reliable measure of visceral fat and overall body fat percentage (Rico-Martín et al., 2020a). In recent years, BRI has been identified as a superior predictor of several chronic diseases, including hypertension (Calderón-García et al., 2021), cardiovascular disease (Cai et al., 2023), gallstones (Wei and Zhang, 2024), metabolic syndrome (Rico-Martín et al., 2020b), and diabetes (Liu et al., 2021). Additionally, BRI has been employed in research to explore its associations with mental health outcomes, including depression, anxiety, and psychological distress (Lotfi et al., 2022). However, the relationship between BRI and suicidal ideation has not yet been investigated. This study seeks to address this gap by examining the association between BRI and suicidal thoughts in a cross-sectional analysis.

## 2 Materials and method

### 2.1 Data source and participants

The data used in this study was extracted from the National Health and Nutrition Examination Survey (NHANES), conducted by the National Center for Health Statistics (NCHS) under the CDC. NHANES aims to evaluate the health and nutritional status of civilians living in the United States through interviews and physical examinations, collecting comprehensive information, including health-related, medical, demographic, and socioeconomic data. The NHANES survey protocol was reviewed and approved by the NCHS Research Ethics Review Board, and signed informed consent was obtained from all participants prior to their inclusion in the survey. This study utilized publicly available anonymized data from NHANES, which is exempt from further ethical review as confirmed by the Ethics Review Board of the University of Galway. Detailed information about the NHANES design, methodology, and data collection is available at http://www.cdc.gov/nchs/nhanes.htm (accessed on the 7th of January 2025).

**Fig. 1:**
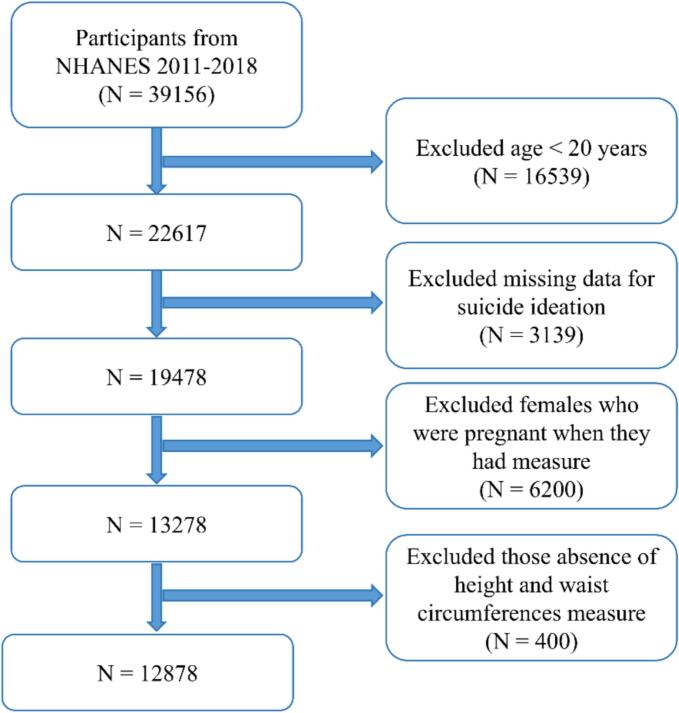
Flowchart of participant selection for U.S. adults aged 20 and above from the National Health and Nutrition Examination Survey 2011–2018.

Given evidence linking the COVID-19 pandemic to suicidal ideation (Farooq et al., 2021), pre-pandemic data was used to reduce potential bias. Initially, the study cohort consisted of 39,156 participants from the NHANES ranging from 2011 to 2012 to 2017–2018. Participants under 20 years of age or with missing suicidal ideation data were excluded from the analysis. Further exclusions were made for participants who were pregnant and those with missing data on height and waist circumference measures. After applying these exclusion criteria, the final analysis included a total of 12,878 participants (Fig. 1).

### 2.2 Assessment of BRI

Body measurement data from the NHANES were collected in the Mobile Examination Centre by trained health technicians, who were assisted by a recorder to ensure the quality and accuracy of the data during the examination process. Additionally, the formula for calculating the BRI was as follows:

$$\mathrm{BRI}=364.2-365.5\times {\left(1-\frac{{\left(\frac{\text{waist circumference}\;\left(\mathrm{cm}\right)}{2\pi }\right)}^{2}}{{\left(0.5\times \text{height}\left(m\right)\right)}^{2}}\right)}^{1/2}$$

where waist circumference was measured in centimeters using a tape measure positioned at the upper edge of the iliac crest, and height was measured in meters (Wei and Zhang, 2024).

### 2.3 Assessment of suicidal ideation

As part of the survey's Mental Health—Depression Screener segment, a vital question “In the past two weeks, how often have you been bothered by thoughts of wanting to be dead or of hurting yourself?” was asked to assess the presence of suicidal thoughts in participants. Answers showing that these thoughts occurred “Nearly every day”, “More than half the day”, and “Several days” were all considered signs of suicidal ideation and were grouped as “Yes”. Participants who reported never experiencing such thoughts were grouped separately as “No”.

### 2.4 Covariates

This study incorporates potential covariates, including demographic factors, lifestyle behaviors, and related chronic noncommunicable diseases, that may influence the relationship between BRI score and suicidal ideation based on prior research. Demographic variables include participants' age (<60/≥60 years), sex (male/female), race/ethnicity (non-Hispanic white, other Hispanic, non-Hispanic black, Mexican American, other), education level (less than 9th grade, 9-11th grade, high school, some college/associate degree, college or higher), marital status (married, widowed, divorced, separated, never married, living with partner), and family poverty income ratio (PIR) (≤1.3, 1.3–1.84, ≥1.85) (National Center for Health Statistics, 2024). Lifestyle factors include smoking status (yes/no), alcohol consumption (≤1 oz., 2–12 oz., ≥13 oz. in the past year), and sedentary time (<600/≥600 min/day). Chronic noncommunicable diseases considered include hypertension (yes/no), diabetes (yes/no/borderline), cancer (yes/no), and cardiovascular diseases (yes/no). Participants were classified as having cardiovascular diseases if they reported being diagnosed with any of the following: heart attack, angina, congestive heart failure, coronary heart disease, or stroke.

### 2.5 Statistical analysis

To examine the demographic characteristics of the participants, the student's *t*-test was utilized for continuous variables presented as mean (standard deviation [SD]), while the chi-square test was utilized for analyzing categorical variables presented as percentages (%). Weighted multivariable logistic regression analysis was employed to examine the association between BRI and suicidal ideation, which involved three models: a non-adjusted model; Model 1 adjusted for demographic factors and lifestyle behaviors; and Model 2 adjusted for all variables in Model 1 plus chronic noncommunicable diseases. Furthermore, weighted stratified analysis and interaction test were used to evaluate whether the relationship between the BRI and suicidal ideation remained consistent across different subgroups. Additionally, smooth curve fitting was conducted to further assess the relationship between the BRI and suicidal ideation. EmpowerStats (version 4.2) (https://www.empowerstats.net/, accessed on September 19, 2024) and R software (version 4.2.2) were used to perform all analyses. A two-tailed *P*-value below 0.05 was regarded as statistically significant.

## 3 Results

The cohort comprised a total of 12,878 participants, of whom 12,437 had never experienced suicidal ideation, while 441 individuals reported experiencing suicidal thoughts. While this study primarily focuses on the relationship between BRI and suicidal ideation, it is essential to consider the potential confounding effects of other key factors.

Table 1 shows that factors associated with a higher likelihood of suicidal ideation included identifying as non-Hispanic White, living alone (widowed, divorced, separated, or never married), having achieved a lower educational level, smoking, and consuming higher amounts of alcohol (≥2–12 oz) in the past 12 months. Furthermore, participants who experienced suicidal ideation were more likely to have lower household incomes (PIR < 1.3) and higher rates of various non-communicable diseases. These was consistent with previous research (Ahmedani et al., 2017; Lange et al., 2023; Poorolajal and Darvishi, 2016; Suicide Prevention Resource Center, 2014). To better understand the independent association between BRI and suicidal ideation, and to control for these potential confounders, a multivariate logistic regression analysis was conducted.

**Table 1:** Characteristics of U.S. adults aged 20 and above from the National Health and Nutrition Examination Survey 2011–2018.

Variables	Without suicidal ideation (*N* = 12,437)	With suicidal ideation (*N* = 441)	*P*-value
Age (%)			0.63
< 60 years	75.6	74.6	
≥ 60 years	24.4	25.4	
BMI, mean (SD)	28.9 (6.8)	29.7 (7.3)	0.02
Sex (%)			0.80
Female	71.8	72.3	
Male	28.2	27.7	
Race/Ethnicity (%)			<0.001
Mexican American	14.1	11.8	
Other Hispanic	9.5	15.4	
Non-Hispanic White	37.1	39.7	
Non-Hispanic Black	22.6	18.4	
Other	16.7	14.7	
Education (%)			<0.001
Less than 9th grade	7.4	10.9	
9-11th grade	12.4	18.8	
High school graduate	22.2	24.7	
Some college/associate degree	31.4	29.7	
College graduate or above	26.7	15.9	
Marital status (%)			<0.001
Married	51.8	34.7	
Widowed	2.7	4.3	
Divorced	8.2	13.8	
Separated	3.0	5.9	
Never married	23.8	30.8	
Living with partner	10.5	10.4	
PIR (%)			<0.001
≤ 1.3	34.9	50.6	
1.3–1.84	14.9	13.6	
≥ 1.85	50.1	35.8	
Smoke (%)			<0.001
Yes	46.0	58.5	
No	54.0	41.5	
Alcohol (%)			<0.001
≤ 1 oz	22.0	16.8	
2–12 oz	77.3	81.4	
≥ 13 oz	0.7	1.8	
Hypertension (%)			0.04
Yes	30.1	34.7	
No	69.9	65.3	
Diabetes (%)			0.02
Yes	11.6	15.9	
No	86.1	82.3	
Borderline	2.2	1.8	
Cardiovascular disease (%)			0.005
Yes	9.0	12.9	
No	91.0	87.1	
Cancer (%)			<0.001
Yes	7.2	11.6	
No	92.8	88.4	
Sedentary time (%)			0.08
< 600 mins/day	81.5	78.2	
≥ 600 mins/day	18.5	21.8	
Waist (cm), mean (SD)	99.55 (16.98)	101.98 (17.68)	0.003
Height (cm), mean (SD)	170.48 (9.30)	170.00 (9.11)	0.29
BRI, mean (SD)	5.26 (2.28)	5.68 (2.56)	<0.001
BRI quartile, (%)			0.003
1.05–3.67	25.1	22.0	
3.68–4.90	25.1	21.5	
4.90–6.37	25.0	24.0	
6.38–20.30	24.7	32.4	

*P*-value was determined by Student's t-test for continuous variables and Chi-square test for categorical variables. Values are presented as mean (SD) or frequencies (%). Abbreviations: BMI, body mass index; PIR, poverty income ratio; BRI, body roundness index; SD, standard deviation.

**Fig. 2:**
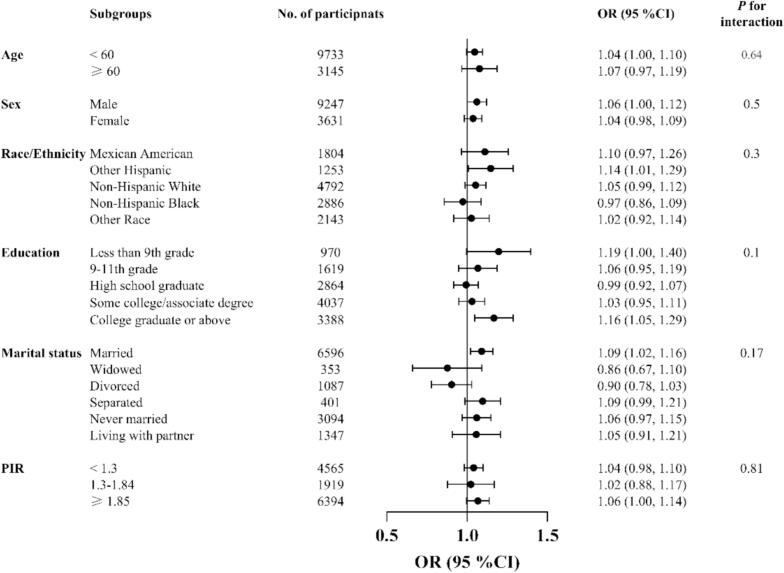
Subgroup analysis assessing the association between body roundness index and suicidal ideation by demographic factors in U.S. adults aged 20 and above from the National Health and Nutrition Examination Survey 2011–2018. *P*-value was determined by weighted interaction test. Abbreviations: PIR, poverty income ratio.

The findings from weighted multivariate logistic regression analyses using three different models are presented in Table 2. A strong and statistically significant positive association between the BRI and the likelihood of suicidal ideation was identified across all three models. According to the unadjusted model, BRI was positively associated with suicidal ideation [1.07 (1.03, 1.11)]. Significant positive associations between BRI and suicidal ideation remained in both Model 1 and Model 2 after adjusting for covariates. In Model 2, a one-unit increase in BRI was associated with a 5 % greater likelihood of having suicidal thoughts [1.05 (1.00, 1.10)]. Additionally, a quartile analysis of the BRI was conducted to further evaluate the relationship, adjusting for all covariates. Participants in the second, third, and fourth quartiles of BRI scores were more likely to report suicidal ideation than those in the first quartile, with all *P* values for the trend being less than 0.05. Notably, participants in the highest quartile of BRI scores were 1.52 times as likely to report experiencing suicidal thoughts compared to those in the lowest quartile [1.52 (1.03, 2.25)]. Moreover, subgroup analyses stratified by demographic information (Fig. 2), including age, education level, sex, PIR, race/ethnicity, marital status and interaction test revealed a relatively stable association between BRI and suicidal ideation.

**Table 2:** Associations between body roundness index and suicidal ideation in U.S. adults aged 20 and above from the National Health and Nutrition Examination Survey 2011–2018.

Exposure	Non- adjusted	Model I	Model II
BRI	1.07 (1.03, 1.11)	1.05 (1.01, 1.10)	1.05 (1.00, 1.10)
BRI quartile			
Quartile 1	1.0	1.0	1.0
Quartile 2	1.02 (0.67, 1.58)	1.18 (0.76, 1.85)	1.17 (0.74, 1.85)
Quartile 3	1.27 (0.87, 1.86)	1.42 (0.92, 2.19)	1.39 (0.88, 2.20)
Quartile 4	1.58 (1.12, 2.23)	1.57 (1.08, 2.27)	1.52 (1.03, 2.25)
*P* for trend	< 0.01	< 0.01	0.02

*P*-value was determined by using weighted multivariate logistic regression. Non-adjusted model adjusted for: None. Model I was adjusted for demographic factors and lifestyle behaviors. Model II was further adjusted for related chronic noncommunicable diseases. Abbreviations: BRI, body roundness index.

## 4 Discussion

In this study, 12,437 participants from the NHANES were used to carry out comprehensive cross-sectional research to examine the relationship between the BRI and suicidal ideation. After taking covariates including demographics, lifestyle factors, and related chronic noncommunicable diseases into consideration, a one-unit increase in BRI was related to 5 % greater likelihood of having suicidal ideation. Moreover, BRI was categorized into quartiles, and suicidal ideation gradually increased with the increased quartile. The positive relationship between BRI and suicidal ideation was linear, which was further confirmed by smooth curve fitting. The robustness of this relationship was shown by stratified analysis and interaction test (*P* > 0.05). To the best of our knowledge, this is the first study to use BRI as a measure to explore the relationship between body shape and suicidal ideation in adult Americans. While prior research has examined the association between body shape and mental health, including suicidal ideation, this study provides new insights into the specific role of abdominal roundness in this context.

One explanation for the observed association may lie in societal attitudes and public health paradigms. Many public health initiatives currently operate within a “weight-normative approach,” which prioritizes weight, weight loss, and weight control as central measures of health and well-being (Mauldin et al., 2022; Tylka et al., 2014). This weight-centric perspective, reinforced by the emphasis on physical appearance and the slender beauty ideal prevalent in westernized societies (López-Guimerà et al., 2010), has led to unintended negative consequences, including restrictive eating behaviors driven by the goal of weight reduction (Neumark-Sztainer et al., 2002). Such behaviors are linked to adverse mental health outcomes, including eating disorders and depression (Kotler et al., 2001; Sharpe et al., 2018), both of which are independently associated with suicidal ideation (Rania et al., 2021; Wang et al., 2020).

Another critical factor to consider is the role of weight-based discrimination, which has been consistently associated with increased suicidal ideation (Hunger et al., 2020). Weight-based discrimination, also referred to as weight stigma, involves prejudiced attitudes, perceptions, and actions that target individuals because of their body size or weight (Westbury et al., 2023). In the United States, nationally representative data suggested that perceived weight discrimination is prevalent, particularly among individuals with higher body weight across sociodemographic groups (Carr and Friedman, 2005). The psychological impact of weight discrimination may be mediated by feelings of perceived burdensomeness, wherein individuals view themselves as a liability to others or as unworthy of support (Brochu, 2020). Furthermore, the intersectionality of weight stigma with other forms of discrimination, such as those based on sex, race/ethnicity, or socioeconomic status, can amplify psychological distress (Puhl et al., 2008).

Internalized weight stigma, where individuals adopt and apply societal negative stereotypes about obesity to themselves (Pearl and Puhl, 2018), represents an additional pathway linking body weight to suicidal ideation. This internalization negatively affects self-image and emotional well-being, leading to feelings of sadness and low self-worth (Pearl and Puhl, 2018), which are strongly associated with suicidal ideation (Brochu, 2020). Notably, weight bias internalization may have even more severe implications for mental health and well-being than experiences of external weight discrimination, though both are undeniably damaging (Brochu, 2020).

From a physiological standpoint, the association between abdominal obesity and suicidal ideation may be explained by several mechanisms, including hormonal dysregulation, insulin resistance, and chronic inflammation. First, increased visceral fat can lead to hormonal imbalances, such as elevated cortisol levels (Mårin et al., 1992), which negatively affect brain function and mood regulation (Genis-Mendoza et al., 2022). Chronic elevation of cortisol has been linked to higher risks of depression and suicidal behavior (Genis-Mendoza et al., 2022). Second, abdominal obesity is closely associated with insulin resistance (Jiang et al., 2020), a condition that disrupts glucose metabolism and has been shown to affect brain function, potentially increasing the risk of depression and suicidal ideation (Watson et al., 2021). Lastly, abdominal obesity is often accompanied by chronic low-grade inflammation, characterized by elevated inflammatory markers like C-reactive protein and interleukin-6 (Bawadi et al., 2019; Yang et al., 2024). These markers have been shown to be associated with both depression and suicidal behavior (Yang et al., 2024), providing a potential biological mechanism underlying the observed relationship between abdominal obesity and suicidal ideation.

Stratified analysis showed the relationship between BRI and suicidal ideation in different subgroups stratified by demographics, and the result of interaction test (*P* > 0.05) demonstrated that this relationship was consistent within subgroups. These reflects the robustness of the relationship, suggesting that at the individual level, routine screening for suicidal ideation in individuals with higher body weights during healthcare visits could be a useful strategy. However, on a societal level, addressing weight stigma may be more effective in mitigating suicide risk. Transitioning from a weight-normative paradigm to a weight-inclusive approach can reduce societal pressures by promoting body diversity, body positivity, and overall health rather than focusing solely on weight loss (Mauldin et al., 2022; Tylka et al., 2014). Anti-discrimination policies, educational programs, and supportive environments are essential to combat weight-based discrimination and alleviate feelings of burdensomeness and psychological distress. Moreover, interventions addressing internalized weight stigma, such as cognitive-behavioral therapy (Pearl et al., 2020) and peer-support groups (Burke et al., 2019), could enhance self-esteem and emotional well-being. For individuals experiencing health challenges related to abdominal obesity, promoting regular physical activity, balanced nutrition, and access to healthcare services may help reduce physiological effects, ultimately lowering suicide risk.

This study is the first to use BRI as a metric to examine the relationship between body shape and suicidal ideation. Utilizing a nationally representative database strengthens the generalizability of the findings. However, this study has several limitations. The cross-sectional design of this study restricts the ability to establish a causal relationship between the BRI and suicidal ideation, allowing only the identification of associations. This study utilized data from the NHANES 2011–2018, which may not fully capture the effects of more recent socio-environmental changes. Although many covariates in demographics, lifestyle factors, and related chronic noncommunicable diseases were selected based on previous research, there may still be the possibility that relevant covariates have not been included. Therefore, future research should consider longitudinal studies to better evaluate the dynamic relationship between the BRI and suicidal ideation. Additionally, integrating qualitative methods and considering underexplored factors, such as psychosocial influences and environmental contexts, may provide more comprehensive insights.

## 5 Conclusion

In conclusion, this study provides novel evidence of a positive association between BRI and suicidal ideation in adult Americans, highlighting the role of abdominal obesity in mental health outcomes. The findings underscore the importance of considering BRI as a potential indicator for identifying individuals at elevated risk of suicidal ideation. The study also emphasizes the need for a shift towards a weight-inclusive approach in public health, which can help reduce societal pressures and mitigate the psychological impact of weight stigma.

## Ethics approval and consent to participate

The survey was approved by the NCHS Research Ethics Review Board, and written consent form was received from each participant.

## Funding

The authors declare that no funds, grants, or other support were received during the preparation of this manuscript.

## CRediT authorship contribution statement

**Pincheng Luo:** Writing – review & editing, Writing – original draft, Software, Methodology, Formal analysis, Data curation, Conceptualization. **Omar M. Alruwaili:** Writing – review & editing, Writing – original draft, Methodology. **Huanlin Zhou:** Writing – review & editing, Writing – original draft, Methodology. **Yanxue Lian:** Writing – review & editing, Writing – original draft, Supervision, Methodology, Conceptualization.

## Declaration of competing interest

The authors declare that they have no known competing financial interests or personal relationships that could have appeared to influence the work reported in this paper.

## Data Availability

Data will be made available on request.
